# Development of an optimised physiotherapist-led treatment protocol for lateral elbow tendinopathy: a consensus study using an online nominal group technique

**DOI:** 10.1136/bmjopen-2021-053841

**Published:** 2021-12-23

**Authors:** Marcus Bateman, Benjamin Saunders, Chris Littlewood, Jonathan C Hill

**Affiliations:** 1 Derby Shoulder Unit, University Hospitals of Derby and Burton NHS Foundation Trust, Derby, UK; 2 School of Medicine, Keele University, Stoke-on-Trent, UK; 3 Faculty of Health, Psychology and Social Care, Manchester Metropolitan University, Manchester, UK

**Keywords:** elbow & shoulder, musculoskeletal disorders, primary care, sports medicine

## Abstract

**Objectives:**

There are a wide range of physiotherapy treatment options for people with lateral elbow tendinopathy (LET); however, previous studies have reported inconsistent approaches to treatment and a lack of evidence demonstrating clinical effectiveness. This study aimed to combine the best available research evidence with stakeholder perspectives to develop key components of an optimised physiotherapist-led treatment protocol for testing in a future randomised controlled trial (RCT).

**Design:**

Online consensus groups using nominal group technique (NGT), a systematic approach to building consensus using structured multistage meetings.

**Setting:**

UK National Health Service (NHS).

**Participants:**

10 physiotherapists with special interest in LET, 2 physiotherapy service managers and 3 patients who had experienced LET.

**Interventions:**

Two consensus groups were conducted; the first meeting focused on agreeing the types of interventions to be included in the optimised treatment protocol; the second meeting focused on specific details of intervention delivery. Participants were sent an evidence summary of available treatments for LET prior to the first meeting. All treatment options were discussed before anonymous voting and ranking of priority. Consensus for inclusion of each treatment option was set at ≥70% based on OMERACT guidelines. Options with 30%–69% agreement were discussed again, and a second vote was held, allowing for a change of opinion.

**Results:**

The optimised physiotherapist-led treatment package included: advice and education, exercise therapy and orthotics. Specific components for each of these interventions were also agreed such as: condition-specific advice, health-promotion advice, exercise types, exercise into ‘acceptable’ levels of pain, exercise dosage and type of orthoses. Other treatment options including electrotherapy, acupuncture and manual therapy were excluded.

**Conclusion:**

An optimised physiotherapist-led treatment protocol for people with LET was successfully developed using an online NGT consensus approach. This intervention is now ready for testing in a future pilot/feasibility RCT to contribute much needed evidence about the treatment of LET.

**Trial registration number:**

This is the pre-cursor to the OPTimisE Pilot and Feasibility Randomised Controlled Trial. Registration: https://www.isrctn.com/ISRCTN64444585

**Strengths and limitations of this study**

- The best available research evidence and stakeholder opinion were combined to develop an optimised physiotherapist-led treatment protocol for people with lateral elbow tendinopathy.
- The intervention was designed for delivery within the UK NHS context and so may need to be adapted to suit other healthcare systems.
- The effectiveness of the optimised physiotherapist-led treatment protocol now needs to be tested in clinical practice.

## Introduction

Lateral elbow tendinopathy (LET), commonly known as tennis elbow, is a painful condition affecting the extensor tendons of the forearm. It is most prevalent in the middle-aged population and therefore can impact on the individual’s ability to work.^1–4^ Point prevalence has been estimated at 1.1%–1.3% of the general population.^3^ For many, it is a condition that resolves over the course of a year, even without treatment, but up to a third of people develop persistent symptoms despite accessing healthcare.^5–10^

There are no established treatment guidelines, although an Australian group of researchers has proffered an algorithm for diagnosis and treatment,^10^ and in the UK, the National Institute for Health and Care Excellence has published a clinical knowledge summary providing advice on management and recommending referral to a physiotherapist.^11^ Physiotherapists offer a wide array of different treatments including advice, exercise therapy, manual therapy, acupuncture, electrotherapies, orthotics and taping.^12 13^ This heterogeneity can be attributed to multiple factors such as variations in training, variations in healthcare funding and personal or patient preference. With wide variations in practice, which include provision of treatments lacking evidence of effectiveness, there is a need to establish an evidence-based, optimum physiotherapy treatment package, to ensure that patients receive the most appropriate treatment in order to improve clinical outcomes for LET. Indeed, even more consistently used treatments, such as exercise therapy, lack a consistent approach to delivery with no consensus on the types of exercise to include, dose of exercise to prescribe and whether exercise should provoke pain or be pain free.^12–16^

Physiotherapy treatment packages are complex interventions involving a combination of verbal and non-verbal communication, patient education and delivery of therapeutic modalities. When designing complex interventions, the purpose should be clear and the intervention should be informed by evidence prior to pilot and feasibility testing.^17^ More recent guidance, from O’Cathain *et al*, encourages stakeholder involvement, including those that deliver the intervention and those that may benefit from it.^18^

This paper reports on the development of an optimised physiotherapy treatment protocol for treating people with LET, using a consensus approach that combined information from a previous synthesis of the best available evidence (see online supplemental file 1) with the perspectives of key stakeholders. The agreed treatment protocol will be assessed in a forthcoming pilot and feasibility trial to determine if it can be delivered in a large-scale randomised controlled trial (RCT).

10.1136/bmjopen-2021-053841.supp1 Supplementary data

## Method

**Figure 1:**
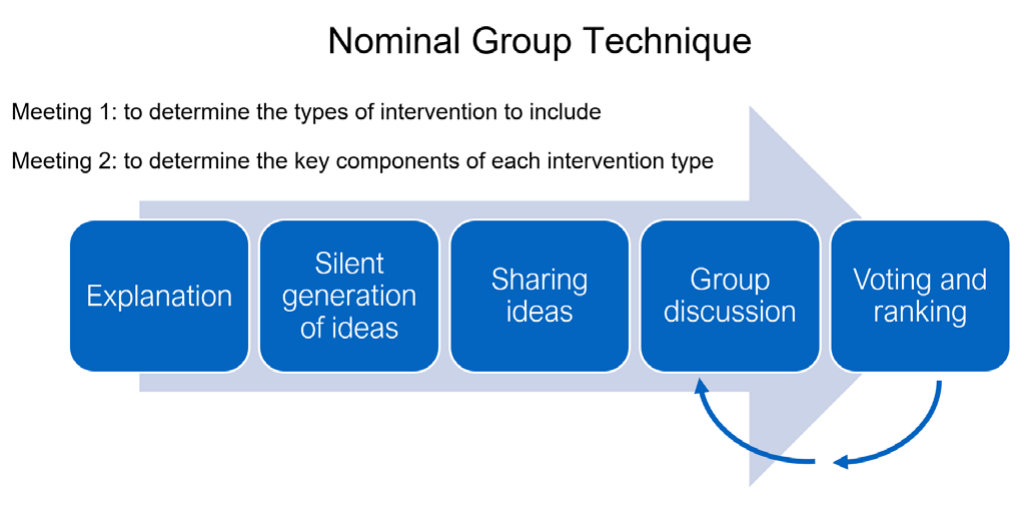
A summary of the nominal group technique process.

The study gained stakeholder consensus for an optimised LET treatment protocol using a nominal group technique (NGT), which is a method that is, by design, dynamic, iterative, creative and open to change. The NGT is usually conducted in face-to-face meetings, about 2 hours long.^19^ For topics that are broad, it is recommended that participants are sent information to read prior to the meeting as a means of pre-elicitation: to facilitate understanding of the NGT process, provide background information (such as a summary of the research evidence of efficacy for physiotherapy treatments for people with LET) and prompt early consideration of the task proposed.^20^ During the meeting, an explanation of the task is then followed by a period of silent idea generation where participants note down their opinions related to the topic or question. These ideas are then shared with the group until no more ideas are forthcoming. There is opportunity to discuss these ideas to gain understanding of individual’s perspectives and clarify definitions, prior to an anonymous vote on whether to include each of the ideas in the final consensus. If voting outcomes are inconclusive, the process can be repeated with further discussion and voting until a conclusion is drawn.^19 21^ The process is summarised in figure 1.

Due to restrictions on face-to-face meeting during the COVID-19 pandemic, the NGT consensus approach was adapted for online data collection with meetings hosted on the Microsoft Teams video-conference platform. Physiotherapists with a special interest in LET were approached to take part via an email advertisement to members of the British Elbow and Shoulder Society and by direct contact with clinicians who had agreed to be part of recruitment and delivery sites for the subsequent pilot and feasibility RCT. Patients volunteered from an existing patient and public involvement and engagement group developed by the research team and physiotherapy service managers were identified from the future trial sites. All participants were required to give written consent to participate, including additional consent to meetings being video recorded.

**Figure 2:**
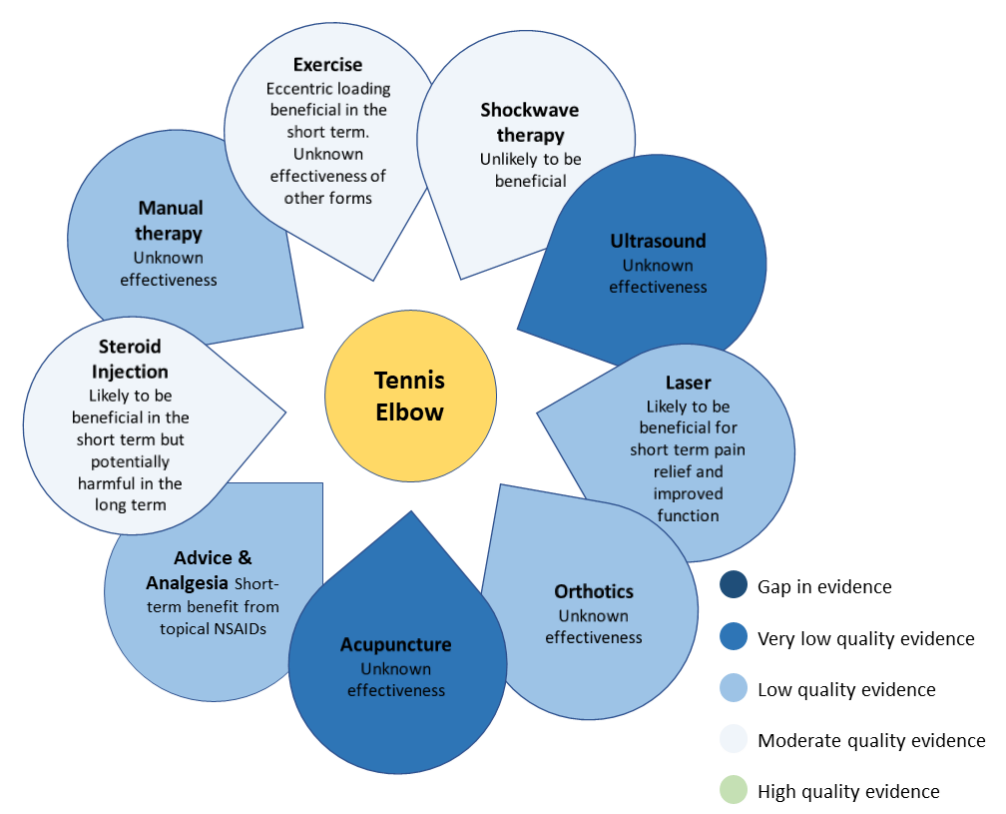
An evidence flower summary of the scientific evidence for the full range of physiotherapy treatments available for people with lateral elbow tendinopathy.

Prior to the first meeting, participants were sent a summary of the evidence synthesis for the full range of LET physiotherapy treatments. The information was summarised in the form of an evidence flower—a visual display designed for conveying the best evidence summaries to professional and lay audiences (see figure 2).^22^ The quality assessment was taken from five previous systematic reviews, the majority of which used the Grading of Recommendations, Assessment, Development and Evaluations (GRADE) system of quality assessment.^14 23–26^ A narrative literature review was also included for those interested in further details about the evidence used (see online supplemental file 1). A comprehensive list of papers was included in the review using systematic search results from a concurrent project, supplemented by hand searching of paper references.^27^ The purpose of the first meeting was to determine the broad types of treatment to include. During the first meeting participants were asked: ‘Which treatments should be included in the optimised physiotherapy treatment package for people with LET?’ They were also asked to consider the evidence presented in the summary documents, whether there were any other treatments that were not in the summary and if any treatments were not feasible for use in their specific UK NHS context. After silent generation of ideas and group discussion, an anonymous vote was conducted using an online voting platform (www.mentimeter.com) with answers only revealed once everyone had voted. Participants were asked to signal ‘yes’ or ‘no’ for the inclusion of individual treatment types in the optimised physiotherapy treatment package. Ratings were averaged across the group, and those with ≥70% agreement (based on the Outcome Measures in Rheumatoid Arthritis Clinical Trials (OMERACT) handbook)^28^ were included. Those with less than 30% agreement were excluded. Treatment types with 30%–69% agreement were discussed further, followed by a second round of voting, to allow for changes of opinion, with those not reaching 70% agreement excluded after the second vote. Finally, the agreed treatment types were anonymously ranked by participants in order of importance using the Mentimeter online platform.

The purpose of the second NGT meeting was to reach consensus on the key components of the treatment types agreed in meeting 1. Prior to the second meeting, participants were sent a summary of the decisions made in the first meeting along with a two-page evidence summary of the component variables related to each of the treatment types selected (for example, the evidence of efficacy for different exercises to be included within the ‘exercise therapy’ treatment). Participants were also encouraged to read the more-detailed narrative literature review to gain a deeper understanding of the evidence available. The second meeting followed the same format as the first, with idea generation, discussion and voting on the individual components to be included within each of the treatment type categories.

### Patient and public involvement statement

Patient representatives with experience of LET were involved in the initial study design, grant funding application and the consensus itself.

## Results

The consensus groups comprised 10 physiotherapists with special interest in LET (mean 18.7 years qualified, range 8–30), 2 NHS physiotherapy service managers and 3 patients (mean age 47). Two of the physiotherapists and one of the managers had also experienced LET themselves. There were eight male participants and seven females. One patient was unable to attend the first meeting due to illness, and all participants attended the second meeting.

**Figure 3:**
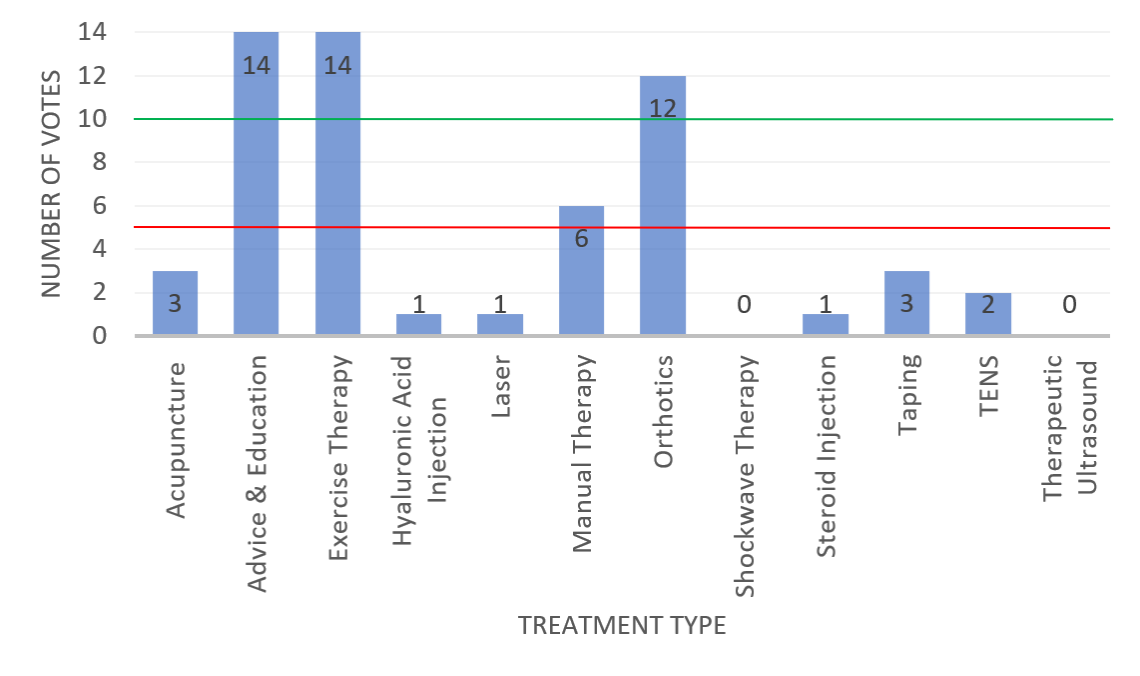
Results of the first voting round from meeting 1—to decide which treatment types will be included in the optimised physiotherapy treatment protocol. Overall, 10 votes were required for inclusion and 5–9 votes required for further discussion and a second vote. TENS, transcutaneous electrical nerve stimulation.

The treatment types proposed and discussed in meeting 1 were: acupuncture, advice and education, exercise therapy, hyaluronic acid injection, laser, manual therapy, orthotics, shockwave therapy, steroid injection, taping, transcutaneous electrical nerve stimulation (TENS) and therapeutic ultrasound. Overall, 14 participants voted on whether to include these treatment types in the optimised physiotherapy treatment protocol, meaning 10 ‘yes’ votes were required to exceed the 70% threshold and 5 ‘yes’ votes required to exceed the 30% threshold. The voting results from the first round of voting are displayed in figure 3. Advice and education, exercise therapy and orthotics surpassed the 70% threshold for inclusion. Manual therapy received 43% of the vote, so was discussed again. Following a second vote, the result remained the same (43%) so manual therapy was excluded. All other treatment types failed to reach the 30% threshold, so were excluded after the initial vote. The three included treatment types were then ranked in order of importance by anonymous vote, with the following outcomes:

1. Advice and education.
2. Exercise therapy.
3. Orthotics.

During the discussion stage of the NGT process, the recommendation from the physiotherapy service managers was that the intervention needed to be adaptable for online consultations, due to recent service changes resulting from the COVID-19 pandemic and future uncertainties around face-to-face consultations in the longer term, and that numbers of follow-up sessions should be minimised to improve efficiency. Patients highlighted the importance of practicality, reducing burden on the patient, and were amenable to online consultation.

In meeting 2, the components of the advice and education treatment were proposed and voted upon. The voting results are shown in table 1.

**Table 1:** Voting results from meeting 2, showing the key components of each treatment category.

Component	Vote 1	Vote 2
Advice and education		
Activity modification	93%*	
Pacing	87%*	
Promotion of self-efficacy	93%*	
Basic pain science	87%*	
Medication advice	80%*	
Sleep advice	47%†	100%*
General exercise advice	80%*	
Stress management advice	53%†	67%‡
Diabetes management	67%†	87%*
Ergonomics for work or sport	93%*	
Smoking cessation	87%*	
What tennis elbow is	93%*	
Diet advice	67%†	100%*
Dietary supplements	N/A	60%‡
Exercise therapy		
Forearm stretches	67%†	80%*
Spine stretches	27%‡	
Isometric loading	93%*	
Concentric loading	93%*	
Eccentric loading	100%*	
Functional exercise	100%*	
Shoulder girdle strengthening	67%†	Grouped and reclassified as ‘Shoulder girdle exercises’
Shoulder girdle stability	80%*	
Shoulder girdle exercises	N/A	80%*
Orthotics		
Counter-force elbow clasp	80%*	
Wrist immobilisation splint	7%‡	
Tubular compression sleeve	13%‡	

*Included.

†Discussed again and revoted.

‡Excluded.

**Figure 4:**
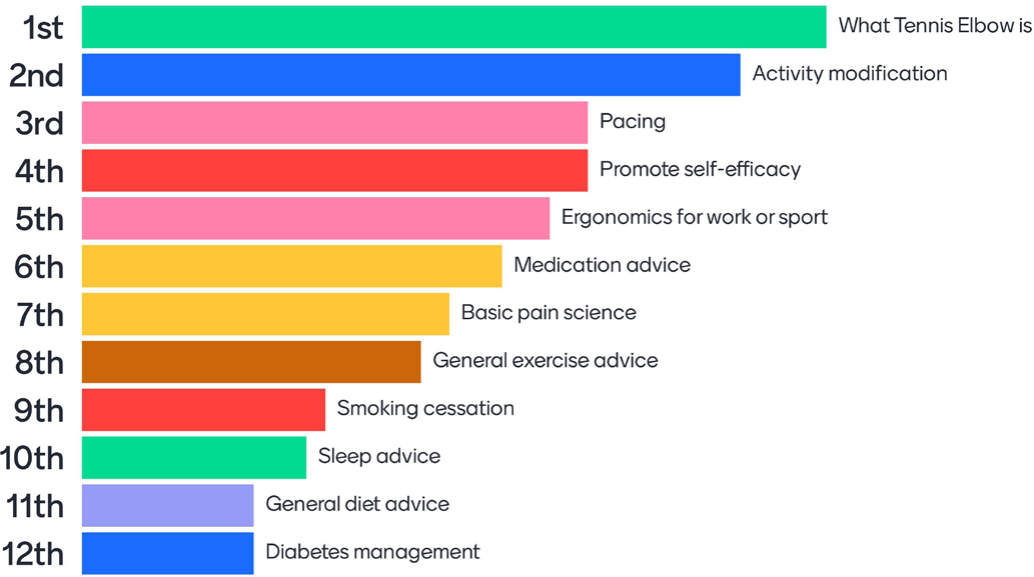
Ranking of included advice and education treatment components in order of importance.

Sleep advice, diet advice, diabetes management and stress management advice failed to meet the 70% threshold but were discussed again and voted upon for a second time. During the discussion, it was agreed among participants that dietary supplements were listed as a separate option for the second vote alongside general diet advice. Following the second vote, only stress management advice and dietary supplements failed to reach the 70% threshold for inclusion, hence were excluded. The full list of agreed advice and education components was: what tennis elbow is, activity modification, pacing, promotion of self-efficacy, ergonomics for work or sport, medication advice, basic pain science, general exercise advice, smoking cessation, sleep advice, general diet advice and diabetes management. The ranking of these components in order of importance is displayed in figure 4.

The components proposed and voted upon for the exercise therapy treatment were: forearm stretches, spine stretches, isometric loading, concentric loading, eccentric loading, shoulder girdle strengthening, shoulder girdle stability exercise and functional exercise. Spine stretches failed to meet the 30% threshold, so were excluded. Forearm stretches and shoulder girdle strengthening were discussed a second time. It was agreed that, on reflection, shoulder girdle strengthening and shoulder girdle stability exercises had significant overlap, so were merged into one category: shoulder girdle exercises. Both forearm stretches (80%) and shoulder girdle exercises (80%) reached the 70% inclusion threshold in a second vote, so the final agreed components were: forearm stretches, isometric loading, concentric loading, eccentric loading, shoulder girdle exercises and functional exercise.

Two further questions were then posed to the participants regarding key components of the exercise therapy intervention:

1. Should exercises provoke pain?
2. What dose of exercise should be used?

Following discussion and voting, it was agreed that exercise should provoke pain to a level that the individual patient deems acceptable to them. Forearm stretches should be held for 30 s and repeated three times before and after loading exercises. Isometric exercises should be held for up to 60 s and repeated five times, once daily. Concentric and eccentric loading should be performed in three sets of 10–15 repetitions, once daily.

For the orthotic treatment, three options were proposed: a counter-force elbow clasp, a wrist immobilisation splint and a tubular compression sleeve. Following voting, the elbow clasp was included (80%) with the other two options excluded (7% and 13%, respectively).

## Discussion

An optimised physiotherapist-led treatment protocol for people with LET was successfully developed using an NGT consensus approach. The agreed intervention consists of (a) advice and education related to both the condition and wider health-related issues, (b) progressive exercise therapy and (c) the provision of an elbow clasp splint. Acupuncture, hyaluronic acid injection, laser therapy, manual therapy, shockwave therapy, corticosteroid injection, taping, TENS and therapeutic ultrasound were excluded.

The NGT consensus approach was easily adapted from the traditional face-to-face format to an online video-conference format without the need for any bespoke software. The online method had the advantage that participants did not have to travel to meetings, allowing for inclusion of a geographically diverse group. A potential disadvantage is that some potential participants could have been put off by the technical aspects of joining a meeting online or lacked the necessary devices, computer skills or internet connectivity.

This study involved a range of different stakeholders (ie, physiotherapists and physiotherapy service managers) that would be involved in future roll-out of the proposed intervention and also patients who would stand to benefit from it. It is hoped that this stakeholder involvement will make the agreed optimised physiotherapy treatment protocol deliverable in a real-life clinical situation. The decision-making process was largely influenced by the scientific evidence, with all of the physiotherapist stakeholders stating that they had read the full evidence review prior to the first meeting; however, the other stakeholders were influential, especially when the evidence was equivocal. Indeed, the input from the physiotherapy service managers shaped the intervention to ensure that all of the elements could be provided via remote online or telephone consultation, should the need arise. Following the result of the first vote in deciding the treatment types to be included, manual therapy was undecided and was discussed again. Some clinicians argued in favour, due to the short-term pain relief that can be achieved with manual therapy, but both the managers and the patients argued against, due to the costs involved with delivering multiple sessions of manual therapy and the burden on the patient of having to attend frequently to receive it. As a result, manual therapy was excluded following a second vote.

The creative nature of the silent generation phase of the NGT process allowed for ideas regarding the advice and education components that differed from previous LET trials. Several trials have included patient education and advice, consisting of explanations of what LET was, reassurance, ergonomic advice, activity modification and medication advice.^5–8^ None, to date, have considered a more holistic approach to health that was reflected in our results, including advice regarding general exercise, smoking cessation, diet advice, sleep, diabetes management and pain science. This has the potential to improve a patient’s overall health alongside influencing the outcome of their LET symptoms.

The components proposed for the exercise therapy intervention were largely in line with previous research evidence. An exception to this was stretching of the cervical and thoracic spine, proposed by four physiotherapists based on their clinical experience, in the absence of any research evidence, but this did not receive sufficient votes for inclusion or further discussion. Forearm stretches were a topic of debate after receiving 67% of the initial vote. Numerous studies have included forearm stretches as part of an exercise therapy intervention alongside strengthening exercises, making it impossible to assess the efficacy of the stretches alone. Only one, three-armed RCT of 94 patients, has compared forearm stretches against the addition of either eccentric strengthening or concentric strengthening.^29^ Outcomes were measured at 6 weeks, with similar effectiveness across all groups. This evidence, along with testimony from two of the participating patients of the immediate pain-relieving effect of forearm stretches, resulted in a change of opinion for the second vote (80%) and inclusion in the exercise therapy treatment.

For the initial exercise therapy vote, shoulder girdle stability exercises had been proposed as well as shoulder girdle strengthening exercises. Following further discussion regarding the details of what participants understood/meant by the two different terms, this resulted in an agreement that there was overlap across the categories and that, overall, a more generic description ‘shoulder girdle exercises’ should be used and included in the exercise therapy treatment. This was largely based on evidence that people with LET have been found to have reduced strength of the shoulder girdle muscles compared with the contralateral arm.^30^

It was agreed that the exercise therapy component should be a progressive regime including a range of exercises to suit patients at different stages of the condition or symptom severity. Previous studies had focused on a single exercise type, for example, isometric loading, finding a plateauing of improvement over time, whereas combined regimes appeared more effective.^8 31^ By including a progressive regime, the aim was to avoid this plateau effect and allow patients to return to their normal level of function.

In a departure from the majority of previous LET studies, this consensus group voted unanimously to include exercises that provoke pain. With the exception of the Stasinopoulos protocol,^32^ which permits exercise into mild pain below 4/10 on a numerical rating scale, all other trials of exercise for people with LET have stated that exercises should be pain free. Pain-related fear can result in higher perceived pain levels due to stress, so an exercise approach that focusses on avoiding pain may exacerbate this response.^33^ Features of sensitisation, such as this hyperalgesia, are a common feature in patients with LET, as identified by 10 studies included in a recent systematic review.^34^ Pain-related fear was recognised as an important factor in this intervention development by all participants, as it could be a mediating variable in the effectiveness of the exercise therapy component. The initial vote was split (47:53%) as to whether to limit pain during exercise to the 4/10 level or let the patient decide how much pain was acceptable to them, but following further discussion influenced by the patient participants the final vote rested in favour of pain to a level that the patient deems acceptable (80%).

The choice of dose for the different exercise types included was largely justified on clinicians’ experience and precedents from particular trials. A systematic review of different types of resistance exercises used to treat people with LET, from 2012, found heterogeneity in the dose of exercise prescribed, with no recommendation possible regarding the optimum dose.^15^ A subsequent systematic review, from 2020, focused just on studies of eccentric loading exercises and recommended that three sets of 10–15 exercises be performed daily, for a minimum of 6 weeks.^14^ This dose was agreed by the consensus group for both eccentric and concentric exercises. The dosing of forearm stretches and isometric exercises was chosen based on what the physiotherapists deemed most pragmatic and the patients deemed most practical/acceptable from examples taken from previous studies showing evidence of efficacy. The agreed dose for forearm stretches was a 30 s stretch performed three times, before and after loading exercises (isometric/concentric or eccentric) as used in the Stasinopoulos protocol.^32^ The agreed dose for isometric exercises was maximal resistance, held for 60 s and repeated five times, as used by Barratt and Selfe.^35^ Two other dosing regimes were considered but the dose prescribed by Park *et al*,^36^ of 50 repetitions of 10 s holds, four times a day was considered too burdensome, and contractions based on percentage of maximum voluntary contraction from 20% increasing up to 35%, used by Vuvan *et al*,^8^ too complicated.

For the orthotic treatment, the decision was between a wrist immobilisation splint, a counter-force elbow clasp and an elasticated elbow sleeve. The latter was proposed as a cheap alternative, but due to a lack of trial evidence to support its use was excluded with just 13% of the vote. The evidence would suggest similar levels of efficacy between wrist immobilisation splints and counter-force elbow clasps.^37–39^ The practicality of such devices was discussed with the counter-force elbow clasps the clear favourite (80%). Reasons given were that wrist immobiliser splints would easily become dirty or wet during work or daily tasks and that elbow clasps were simpler to provide and stock, as they are universal in terms of fitting the left or right arm and have fewer sizing options than wrist immobilisation splints.

The main strength of this study is that a clinical trial intervention protocol has been developed using the combination of the best available research evidence and stakeholder opinion. The optimised physiotherapist-led treatment protocol was designed to be deliverable in the UK NHS, but could be adapted to suit other healthcare systems. Other strengths were: the inclusion of multiple voting rounds to allow for discussion and change of opinion in light of new information and the use of the evidence synthesis to guide decisions based on the evidence base, which the study used a recommended consensus approach, and that voting thresholds were consistent with established OMERACT guidelines. A limitation is that it is based on evidence available at the time of the event and the opinions of those involved in the process. The decisions were largely based on scientific evidence but were influenced, particularly in cases where evidence was equivocal, by an individual’s experience. It must also be noted that the effectiveness of the optimised physiotherapist-led treatment protocol still needs to be assessed against usual physiotherapy care before it can be recommended for use in a clinical setting. Funding and ethical approvals are in place to test this in a feasibility trial involving 50 participants.

## Conclusion

This study successfully developed an optimised physiotherapist-led treatment protocol for people with LET, which was considered feasible by stakeholders and adaptable for use in online consultations, if required. It includes advice and education related to the condition and the patient’s general health, progressive exercise therapy that provokes a pain response and the provision of an elbow orthosis. This intervention is now ready for testing in a future pilot RCT to contribute much needed evidence about the treatment of LET.

## Data Availability

Data are available upon reasonable request.
