# Supplementary material for: Development of an optimised physiotherapist-led treatment protocol for lateral elbow tendinopathy: a consensus study using an online nominal group technique
Source: BMJ Open. 2021 Dec 23;11(12):e053841. doi: 10.1136/bmjopen-2021-053841 (PMC8712984; doi:10.1136/bmjopen-2021-053841)
Supplement: Supplementary data [file bmjopen-2021-053841supp001.pdf]

Literature Review of Physiotherapy Interventions for Lateral Elbow Tendinopathy

M Bateman[1,2], B Saunders[2], C Littlewood[3], J C Hill[2].

1. Derby Shoulder Unit, University Hospitals of Derby & Burton NHS Foundation Trust
2. School of Medicine, Keele University
3. Faculty of Health, Psychology and Social Care, Manchester Metropolitan University

Correspondence: Marcus Bateman, Derby Shoulder Unit, Royal Derby Hospital, Derby, DE22 3NE. email: marcus.bateman@nhs.net

Initial Management Strategies

The NICE Clinical Knowledge Summary[1] suggests that initial management should recommend the use of analgesia such as paracetamol or topical non-steroidal anti-inflammatory drugs (NSAIDs), with a subsequent prescription of oral NSAIDs if ineffective. It is recommended to give advice to avoid heavy lifting, avoid forceful gripping and twisting activities, favour palm-up lifting rather than palm-down, and modify work by taking more rest breaks, alter work patterns and change practice regarding lifting.

Evidence for simple advice

Similar advice has been used as part of a wait-and-see control arm in five trials, along with simple reassurance that for the majority the symptoms of LET will settle over time.[2-6] In all five, patients in the wait-and-see group improved with short-term patient-rated successful treatment ranging from 26.3% to 48% and longer-term success at one year ranging from 75% to 90%. It is unclear whether this represents the natural course of the condition or whether the advice improved outcomes, given that there have been no studies of advice versus a true wait-and-see approach.

Epidemiological studies suggest that there may also be a place for advice related to stopping smoking, improving diabetes control and promoting regular exercise two to three times per week based upon risk factors for developing the condition.[7,8]

The Kings Fund, in 2015, set ten priorities for UK NHS commissioners that included self-management at number one, with the aim of promoting increased physical function and self-confidence.[9] Self-management "refers to activities which promote health but also prevent deterioration by gaining skills which can be applied to new problems as they arise to increase self-efficacy in managing the condition as it progresses."[10] Systematic reviews of the musculoskeletal literature, whilst not specific to LET, show moderate to strong evidence for the use of exercise and psychological interventions, such as pain coping skills, as physical activity and pain catastrophising are strong mediators for outcome in studies of self-management.[11-13] It is recommended that self-management education is delivered to patients by healthcare clinicians and includes follow-up sessions rather than one-off advice, should include self-help materials, help patients to identify problems specific to themselves, assist the patient to form personalised coping strategies and enhance their self-efficacy by empowering them to take responsibility for their lifestyle choices.[10,14,15] Applying such methods, in addition to the basic advice given in the LET trials previously mentioned, may further improve outcomes.

Sport-related advice

In racquet-sports players, it has been hypothesised that changing grip size on the racquet may help to reduce symptoms by altering the grip force required to hold it, but a laboratory study found no difference in muscle activity with different grip sizes.[16] Racquet string tension has though been found to relate to changes in force transmission across the elbow during backhand tennis groundstrokes, with higher string tension resulting in higher force.[17] Similarly, a tighter grip on the racquet combined with below-centre strikes on the racquet face result in higher eccentric wrist extension torque.[18] Whilst these two studies were performed in laboratory experimental conditions, the biomechanical findings could be transferrable to real-world sport with advice to de-tension strings, grip the racquet less firmly and seek coaching to improve ball-strike technique.

Evidence for the use of analgesia

Systematic review evidence of five placebo-controlled trials investigating the use of topical NSAIDs suggests that this can offer short-term pain relief up to four weeks but the evidence was judged to be of low quality and therefore inconclusive.[19] The evidence for oral NSIADs was conflicting. No trials have specifically investigated the use of paracetamol or opioid medication though it stands to reason that these may offer short-term symptomatic pain relief only rather than affecting the overall course of the condition, as found with other musculoskeletal disorders, such as back pain and shoulder pain.[11]

Evidence for use of physiotherapy interventions

In this section the evidence for these treatments will be analysed and discussed:

Manual therapies

Manual therapy includes a range of different 'hands-on' treatment techniques that, in the case of LET, can be grouped into Cyriax manual therapy, Mobilisation with Movement (MWM) and regional mobilisations.[20] The Cyriax method involves a 10-minute session of deep transverse friction massage to the painful tendon followed by a Mills' Manipulation whereby the patient's elbow is forcibly extended to end range whilst the wrist is fully flexed and the forearm pronated.[21] MWM combines manual therapy with active exercise, typically a lateral glide to the elbow whilst the patient performs an isometric gripping exercise.[22] Regional mobilisations include all other types of manual therapy used more generally in the upper limb, rather than focussed on the elbow, and mobilisation of the cervical spine.[20]

The most-recent systematic review and meta-analysis of manual therapy for LET by Lucado et al[20] concludes that "there is compelling evidence that joint mobilizations directed at the elbow improve both pain and functional grip scores across all time frames compared to control groups in the management of LET." This conclusion must, however, be questioned based upon methodological errors and reporting bias in the review. Three large studies are included in the meta-analyses that investigate manual therapy as part of a multimodal physiotherapy treatment package compared with a control of wait-and-see (including advice).[2,6,23] It is impossible to determine the effect of the manual therapy component of these studies which should not have been included in the meta-analyses for that reason. With these studies removed the meta-analysis of Mills' Manipulation (Cyriax manual therapy) would not be possible for pain as only one study would remain. The meta-analysis of pain for MWM would only include one small pilot study of 10 patients and a small non-randomised study of 34, with no analysis possible for follow-up beyond four weeks.[24,25] Grip strength would not be possible as only one study would remain.[24]

Reviewing the remaining evidence descriptively, Cyriax manual therapy is no more effective than Bioptron polarised light therapy based upon no significant difference in any outcome measures or time points apart from pain visual analogue scale (VAS) at 28 weeks.[26] The same study included an exercise intervention arm and found that exercise was more effective than Cyriax manual therapy at all time points and all outcome measures up to 28 weeks.[26] Similarly, Viswas et al[27] compared Cyriax manual therapy against the same exercise intervention designed by Stasinopolous[28] and found similar results in favour of exercise. In contrast, Nagrale et al[21] found Cyriax manual therapy to be superior to a combination diclofenac gel phonophoresis and Stasinopoulos exercises at eight weeks.

Two studies have investigated the immediate effect of MWM on pain free grip strength (PFGS) and pressure-pain threshold (PPT) after a single treatment session.[22,29] The studies were small, totalling 41 patients, but had robust methodologies that included a placebo and control procedure, and blinded both the patient and the outcome assessor to the intervention. Both found significant immediate improvements in PFGS compared to a sham MWM group and a no intervention group. There are few studies, however, that investigate longer-term effect: two studies investigated the addition of MWM to multimodal physiotherapy including heat, massage and ultrasound therapy. Amro et al[24], in a study of 34 patients, found in favour of the MWM group at four weeks follow-up but the method was non-randomised and at high risk of bias. Kim et al[25] also concluded that the addition of MWM improved outcome immediately after 10 days of treatment but with just 10 patients the study was under-powered. Afzal et al[30] found that patients treated with MWM and ultrasound therapy had significantly improved pain and function at four weeks follow-up compared to those treated with ultrasound alone but the study was limited by a small sample size (n=30) and a lack of blinding. A novel study by Martinez-Cervera et al[31] investigated the mechanism by which MWM might have an effect. Twenty-four patients were randomised into two groups that both received MWM three times in a week. Half of the patients were told that MWM was a very effective treatment and the other half were given neutral expectations that it may or may not be effective. Patients given high expectations gained significantly better outcomes immediately after treatment suggesting that patient expectation might be an important factor in treatment selection.

Regional mobilisations can be divided into wrist mobilisation and cervical spine mobilisation. The evidence for wrist mobilisation is limited to two small un-blinded studies of similar methodology compared against multi-modal physiotherapy.[32,33] Both found short-term benefit in favour of wrist mobilisation at three weeks but Struijs et al[33] also followed-up patients to six weeks and found no difference between groups at that time point. The evidence for cervical mobilisation is based upon three small randomised trials totalling 43 patients and one low-quality retrospective study.[34-37] Vicenzino et al found immediate improvements in PFGS, pain VAS and PPT with mobilisation of the C5/6 cervical levels compared to a sham technique or control.[37] Fernandez-Cervaro et al conducted two studies where cervical manipulation was firstly compared with a sham technique and secondly compared with thoracic manipulation.[35,36] Both reported immediate improvement in PPT but conflicting results for PFGS. The retrospective study by Cleland et al[34] concluded that there was a high long-term success rate for multimodal physiotherapy with or without cervical mobilisation. Small differences were seen in favour of cervical mobilisation group but given that the patient demographics and treatments received as part of the multimodal physiotherapy between groups were different the attribution of this effect to manual therapy alone is unjustified.

Overall, there is low quality evidence to suggest short-term benefit of manual therapy but also that it may be less effective than exercise.

Orthoses and Taping

Orthoses

Orthoses for LET are widely available for general public sale and are also provided via the UK NHS on the recommendation of clinicians.[38] Different forms of orthotics are available but the two main principles of treatment are either to immobilise the wrist, thus reducing the activity of the wrist extensor muscles, or to alter the mechanical forces along the extensor muscles of the forearm by use of a 'counter-force brace'. Counter-force bracing involves fastening a tight cuff around the forearm containing a padded section that is sited over the ECRB muscle. Cadaveric studies have shown that this reduces the force on the ECRB tendon origin when a load is applied distally, suggesting that *in vivo* the aggravating load on the ECRB might be reduced when performing gripping activities whilst using the brace.[39] This has been demonstrated in a small LET patient population where 31 patients were randomised to either wear the brace correctly as a tight cuff or to wear it loosely to minimise the effect.[40] Those wearing the brace correctly experienced significant pain relief in the short term compared to those wearing it loosely. Likewise, a cross-over study investigating two different types of counter-force brace (one a standard design and another incorporated into an elbow compression sleeve) found that these gave immediate pain relief and improved grip strength compared to no brace.[41]

The use of a wrist immobilising splint has been shown to improve pain and grip strength after three weeks when used in combination with physiotherapy treatment and compared to physiotherapy treatment alone.[42] Two studies have compared the use of counter-force bracing to wrist immobilisation, with different conclusions drawn: Akkurt et al[43] found no difference between the different types of splint up to six weeks follow-up of 82 patients whereas Garg et al[44] concluded that wrist immobilisation was superior at the same time point when studying 42 patients. This conclusion is questionable however, as it was only demonstrated in one sub-domain of the American Shoulder and Elbow Society (ASES) Elbow Assessment Form when all other outcome measures showed no difference. Both studies showed that patients with LET improved over time regardless of which orthosis was used. Van De Streek et al[45] compared the use of a counter-force brace to both the counter-force brace and wrist immobiliser worn together and found no difference in outcome between groups at six weeks.

Whilst there is some evidence of short-term effect of orthotic use, there may be no effect in the long-term. A large study of 110 patients with LET by Nishizuka et al[46] compared a counter-force brace worn daily for six months in addition to exercises with exercises alone. There were no differences in outcomes between groups at any time point up to one year, but both groups improved significantly suggesting the brace gave no additional benefit to exercises alone. Similarly, a large study of 185 patients compared the use of a counter-force brace against an exercise programme and found in favour of exercise at all time points up to a minimum of 12-month long-term follow-up.[47] Indeed, a large retrospective population study of 4614 patients receiving treatment for LET and medial elbow tendinopathy (MET) in the USA found that those using orthoses of any type had higher healthcare usage, longer treatment duration and longer time off normal work than those that did not use orthoses. Other factors may though confound this conclusion as it was unclear whether the baseline symptoms (such as pain severity) were similar between those using orthoses and those not. Higher baseline pain is an established predictor of poorer outcome in patients with LET[48] so the differences between groups may not be due to orthotic use alone.

Taping

Kinesiology tape (or K-tape) is an adhesive elasticated tape that is purported to reduce the load on the wrist extensor tendons when applied longitudinally over the dorsal forearm muscles.[49,50] It is not

commonly used in UK practice.[38] Studies of the use of K-tape to treat LET are of low quality and of small sample size.[49,51-55] Cho et al[50] found that the application of K-tape to patients with LET gave some immediate pain relief for up to 15 minutes but for longer follow-up the majority of studies show that the use of K-tape is no more effective than sham taping techniques or offers no increased benefit when used in addition to other physiotherapy modalities such as exercise.[49,51,54] The exception is a study by Giray et al[55] but with only 10 patients per group the result may have been due to chance.

Diamond taping uses a non-elastic adhesive tape applied in four strips pulled tightly around the location of lateral elbow pain to form a diamond, resulting in the encompassed skin having an orange-peel appearance.[56] Similarly to K-tape it is purported to reduce mechanical load on the wrist extensor tendons.[56] A recent systematic review identified four studies of diamond taping each only measuring the immediate effect after application or up to 30 minutes afterwards.[57] All four studies showed improvements in either pain or grip strength compared to controls. It is unclear however whether this has any useful clinical benefit as longer-term effects have not been studied.

Acupuncture

Acupuncture is used by some physiotherapists in the UK as a second-line treatment for LET.[38] It involves the insertion of fine needles into specific anatomical points on the body as defined in Traditional Chinese Medicine (TCM). These points are then stimulated in a variety of ways such as by twisting the needles (manual stimulation), applying an electrical current (electro-acupuncture) or by heating the needles (moxibustion).[58,59] The purpose is to induce a pain-relieving effect on the nervous system although the evidence for this effect has not been firmly established.[59]

The evidence for the use of acupuncture in the treatment of LET is of low or very low quality based upon several systematic reviews.[58,60-62] Of the included studies, only four compare acupuncture with a supposed placebo or sham treatment. It might be argued, though, that in three of these studies the control arm still included acupuncture treatment: Fink[63] and Irnich[64] both used a similar method whereby acupuncture needles were still inserted but at least 5cm away from the sites recommended by TCM; in the study by Haker[65] needles were still inserted at acupuncture sites but only superficially rather than to the recommended depth, and were not stimulated. In the fourth study, Molsberger[66] used a sham control method where pressure was applied to an acupuncture point on the patients' thoracic spine with a pencil-shaped probe instead of a needle being inserted but patients could not be blinded from this as the 'real' acupuncture group did not have any needles inserted in the thoracic region. Despite this, in all four of these studies outcomes favoured 'real' acupuncture immediately post-treatment or up to two weeks' follow-up. A limitation of the majority of acupuncture studies is the lack of longer-term follow-up, lack of blinding, lack of randomisation and heterogeneity of outcome measures that prevents meta-analysis of data.[58] Few studies measure the impact on disability and function, just focussing on pain severity.[62] Fink[63] and Haker[65] both followed-up patients for one year but no significant differences were seen between 'real' acupuncture and sham acupuncture beyond two weeks. Improvements were seen in both groups following the natural trend for improvement in LET symptoms over time. The evidence for acupuncture treatment for LET is therefore uncertain but it may offer some short-term benefit for pain for up to two weeks.

Electrotherapies

Electrotherapy was established as one of the four pillars of UK physiotherapy practice when the Society of Physiotherapy was granted its Royal Charter in 1920. Over the century that followed electrotherapies changed with evolving technology but the principle of the purported mechanism of effect remained the same: when energy is focussed on injured tissue it can improve the healing response.[67-71] Electrotherapy is still used in the management of LET in the forms of laser, ultrasound and shockwave therapy (SWT).[38,72]

Laser

Laser treatment uses light energy applied locally to the area of pathology to stimulate a physiological response such as reducing inflammation or promoting collagen production.[73] The reaction is dose-dependent with collagen production at lower doses and anti-inflammatory effects at higher doses.[73] For this reason Low Level Laser Therapy (LLLT) is most commonly used in the treatment of LET to promote collagen repair in the absence of significant inflammation.[74] Laser light can be generated at different wavelengths dependent on the elements used: gallium arsenide 904nm, helium neon 632nm, gallium aluminium arsenide 820nm and neodymium-doped yttrium aluminium garnet 1064nm.[67,75,76] These different wavelengths penetrate human soft tissues differently with 904nm having the deepest effect.[77] The use of laser was popular in the 1990s but has since declined in both usage and availability.[78] Recent studies of UK practice showed that it was now scarcely used in the treatment of patients with LET.[38,72]

A systematic review of the effectiveness of LLLT in the treatment of LET published in 2008, Bjordal et al[73] concluded that it offered favourable short-term improvements in both pain and function when compared to placebo. In a previous review, Bisset et al[79] had concluded that laser was no more effective than placebo but in this study the analysis was not broken down into different treatment wavelengths. Bjordal et al[73] sub-classified studies by treatment wavelength in their meta-analysis to find that the 904nm wavelength provided an effective response (when applied over the extensor tendons rather than when applied over acupuncture points) immediately after the course of treatment and up to eight weeks of follow-up. The 820nm and 1064nm showed no benefit and the 632nm wavelength was inconclusive but might be effective based upon one study.[80]

Ultrasound

Ultrasound therapy delivers energy locally to the tissues via high frequency sound waves. The evidence for ultrasound is conflicting and of low or very-low quality.[60,61,71,81] Smidt et al[81] in a systematic review published in 2003 pooled data from two studies to conclude that ultrasound was effective for pain relief in the medium-term up to 13 weeks but the studies were low-powered. Indeed, considered separately these two studies show conflicting results: Binder et al[82] demonstrated significant benefit from ultrasound over placebo whereas Lundeberg et al[83] found no difference. A subsequent study of similar methodology comparing ultrasound against placebo also found no difference in outcome.[69] Subsequent reviews in 2014 and 2015 have concluded that ultrasound is no more effective than placebo in the short-term.[71,84] However, Dingemanse et al[71] still concluded that there was moderate evidence in favour of ultrasound over placebo in the medium-term despite this being based on the outcome of just one study that could not be replicated.

Shockwave therapy

Shockwave therapy provides energy to the tissues via pulsed acoustic waves, but the mechanism of any therapeutic effect is unclear.[70] Shockwave therapy can be administered in different ways: by use of a radial shockwave device or an extracorporeal shockwave device, and with or without the

addition of local anaesthetic. One method has not been shown to be superior to the others.[84] The continued clinical use of SWT is surprising given the conclusions of a 2006 systematic review stating that based upon "platinum-level evidence that shock wave therapy provides little or no benefit in terms of pain and function in lateral elbow pain."[70] A more recent review published in 2015 pooled data from the 2006 review with subsequent studies to draw similar conclusions: that SWT was no more effective than placebo for pain or pain on resisted wrist extension up to six weeks follow-up.[84] Despite this, it continues to be used in UK practice for the treatment of LET by 11% of respondents to a recent nationwide survey.[38]

Exercise therapy

Exercise is the mainstay of modern physiotherapy treatment of LET in the UK.[38,72] A limitation of the evidence regarding exercise is the heterogeneity of exercise type, treatment duration and dosage used in clinical trials.[85] Many trials have used bespoke exercise programmes but there are four specific exercise protocols have been studied multiple times:

The Pienimaki protocol

The Pienimaki protocol was first described in 1996 in a trial of exercise versus ultrasound therapy.[86] It consisted of stretches of the forearm muscles and a four-stage progressive loading regime starting with isometric contractions, then isotonic resisted uniplanar exercises using a Theraband, followed by isotonic resisted biplanar exercises using a Theraband, and finally functional repetitive movements involving gripping. Patients were advised to perform exercises four to six times per day for six to eight weeks. Each exercise was done in two to three sets of 10 repetitions. The findings of the trial showed that the exercise protocol was significantly more effective than ultrasound immediately after eight weeks of treatment.

The same exercise protocol was subsequently used with deep transverse friction massage and ultrasound as part of a multimodal physiotherapy treatment package by Smidt et al.[4] The multimodal package gave the highest chance of recovery at six months compared to corticosteroid injection or wait-and-see.

It was also used by Tonks et al[87] in a low-powered randomised controlled trial (RCT) involving 12 patients per group. Improvements were seen at seven-week follow-up in pain and grip strength compared to controls but failed to reach statistical significance.

The Stasinopoulos protocol

Stasinopoulos et al[26,28] described a four-week supervised exercise protocol consisting of one stretching exercise and a progressive eccentric loading exercise. A stretch of the wrist extensor muscles was performed with the elbow extended, forearm pronated and wrist passively flexed with ulnar deviation to the end of the available range. The position was maintained for 30-45 seconds and repeated three times before and after the eccentric loading exercise. Eccentric loading was performed with the elbow fully extended and forearm pronated whilst supported on a treatment couch. The wrist was passively positioned into full extension then slowly lowered to full flexion over 30 seconds with the addition of a load individualised to the patient. The load was applied using a weight or Theraband and determined by the pain response. Mild pain was acceptable but disabling pain meant that the load was too great. Eccentric exercises were performed in three sets of 10 with a one-minute rest period in-between sets.

The Stasinopoulos protocol has been used in seven trials.[21,26,27,88-91] It has been compared to the Pienimaki protocol and found to give greater benefit in terms of pain relief and function at 12 and

24-week follow-up.[90] Patients performed supervised exercises once per day, five days per week for four weeks compared with home exercises four to six times per day for eight weeks in the Pienimaki protocol. Adherence to home exercise was not measured but the authors hypothesise that adherence may have been the deciding factor in why the Stasinopoulos protocol was more effective. An alternative reason could be the different types of exercise used.

Three studies have compared the Stasinopoulos protocol to Cyriax manual therapy.[21,26,27] The two studies that used the protocol as a stand-alone treatment found it to be superior to Cyriax manual therapy[26,27] but Nagrale et al[21] combined it with diclofenac gel phonophoresis and found it to be less effective.

Manias et al[88] investigated whether the addition of ice massage to the exercise protocol was more effective than the exercises alone and found no difference in outcome. Both Sethi et al[89] and Mostafaee et al[92] added shoulder strengthening exercises to find that these further improved outcomes when compared to the Stasinopoulos protocol alone. Likewise, the addition of concentric and isometric strengthening exercises resulted in superior short-term results when compared to the original protocol.[91]

The Solveborn protocol

The Solveborn protocol[47] consisted of 10-second isometric wrist extension contractions followed by stretches of the forearm extensor muscles held for 15-20 seconds. Isometric contractions were performed three to five times followed by a similar number of stretches. Then, similar exercises were performed for the wrist flexors. Pain during exercise was avoided. Exercises were performed twice daily. In a large trial of 185 patients, the exercise protocol was compared with the use of a counterforce brace. Both groups improved but the exercise group had significantly better outcomes at all time points up to and beyond a year follow-up.

The protocol was used in three other trials.[93-95] Nilsson et al[94] taught the exercise protocol for home use along with ergonomic advice and a counterforce brace in a non-randomised trial versus a control of usual care. The intervention group had significantly better outcomes at four and 16-week follow-up but there was a high drop-out rate in the control group that may invalidate the results. Haahr et al[93] conducted a large RCT involving 266 patients randomised to a one-off education session, including general advice and instruction in the Solveborn protocol, versus a control group of usual care. They found that both groups improved up to one year but with no between-group difference. Svernlov et al[95] compared the Solveborn protocol to a combination of stretching and progressive eccentric loading. The same stretching dose was used but the isometric exercises used in the Solveborn protocol were substituted with three sets of five repetitions of pain-free eccentric loading exercises using a weight. Each repetition was performed over 10 seconds. The weight was progressively increased by 10% each week from a starting point of 1 kilogram for males and 0.5 kilograms for females. Both groups exercised at home for 12 weeks. Improvements were seen in both groups but the eccentric exercise group gained significantly improved grip strength at six months compared to the Solveborn protocol group.

The Vicenzino protocol

The Vicenzino protocol[96] has been used in three large RCTs totalling 483 patients.[2,23,97] In all three trials it has been used as part of a multimodal approach along with manual therapy and taping. The exercise component required patients to perform pain-free exercises of the hand, wrist and forearm starting with simple controlled active movements not incorporating additional load. Load was then progressively added using Therabands of increasing resistance during concentric and eccentric actions of the wrist. The focus was on wrist extension with exercises performed slowly over six to

eight seconds. The dose was dependent on the symptom reaction with pain avoided during and after the exercises. As symptoms improved with gripping no-longer painful, additional strengthening exercises of the whole upper limb were prescribed including bench press, shoulder press, bent-over rows, biceps curls and tricep curls using weights. In two trials patients attended eight times over six to eight weeks[2,23] and in one trial four times over four weeks.[97] Significant improvements were seen between four and 26 weeks follow-up across the trials compared to controls and economic evaluation from the trial by Coombes et al found it to be a cost-effective treatment for LET.[98]

Isometric exercises

Isometric exercise as an initial treatment for the management of acute tendinopathy is currently *en vogue.*[99] Two studies have investigated isometric exercises specifically for the treatment of LET.[5,100] Park et al[100] randomised 31 patients to early pain-free isometric wrist extensions or the same exercises started after four weeks. The contractions were held for 10 seconds and repeated 50 times, four times a day. Significant improvements were seen in the first four weeks in the early exercise group. Vuvan et al[5] compared a single session of isometric exercise instruction versus wait-and-see in a trial of 40 patients. Patients were taught to perform the exercises at 20% of the Maximum Voluntary Contraction (MVC) of the unaffected arm increasing to 35% MVC by week seven. They performed three repetitions of 45 second holds or four repetitions of 30 second holds once daily for eight weeks. Outcomes measured using the PRTEE improved significantly in exercise group at eight weeks but other measures did not show a significant difference. The authors concluded that isometric exercise alone was not sufficient to treat LET but may form part of a treatment package.

Stasinopoulos et al[91] compared their own protocol of eccentric and stretching exercises to the addition of concentric exercises and both concentric and isometric exercises. A small and insignificant difference was seen with the addition of concentric exercises but the further addition of isometric exercises resulted in significant improvements compared to eccentric and concentric/eccentric exercises. The study was, however, limited by a small sample size of 34 so the results should be taken with caution.

Eccentric exercises

The most commonly studied form of exercise for LET is eccentric exercise.[85] A 2020 systematic review by Chen et al[101] showed a large effect of eccentric exercise over other treatment modalities or other forms of exercise but noting that in many studies the eccentric exercise was used as part of a multimodal treatment. There are several studies though that have investigated eccentric exercise in isolation. Tyler et al[102] compared a multimodal approach with and without eccentric exercise using a Theraband Flexbar device. It was a small study of 21 patients but the addition of eccentric exercises significantly improved outcomes after six weeks of treatment. The same technique was used by Tiwari[103] and compared to concentric and eccentric exercises using a weight, performed daily. After the three weeks of treatment outcomes significantly favoured the Theraband Flexbar technique but the difference may be attributable to dosing rather than technique as patients using the Theraband Flexbar performed 45 repetitions per day compared to 20 repetitions in the other group.

In contrast, Martinez-Silvestrini et al[104] compared stretching against stretching with the addition of either concentric or eccentric exercises. They found that all groups improved a similar amount at six-week follow-up although the eccentric exercise group suffered fewer exacerbations of symptoms.

Soderberg et al[105] treated 42 patients using a counterforce brace with and without the addition of eccentric wrist extension exercises. A simple method was employed where patients exercised at home holding a bucket with increasing amounts of water to increase load. After six weeks of follow-up the group performing eccentric exercises had significantly better outcomes.

A higher quality study by Crosier et al[106] randomised 92 patients to a multimodal physiotherapy treatment package of ice, TENS, ultrasound and stretching exercise versus multimodal physiotherapy plus eccentric exercises. The eccentric exercises involving wrist extension and forearm supination were performed using a Cybex isokinetic machine three times a week for a total of 25 to 26 sessions. Two sets of 10 exercises were performed for each movement with gradually increasing velocity and resistance over the treatment period up to 90° per second and 80% MVC. Significantly improved results were seen in the eccentric exercise group at the end of treatment but the practicality of an intervention requiring high levels of patient attendance must be questioned.

Other exercise protocols

Peterson et al[3] used a similar method to Soderberg et al[105] teaching patients to exercise at home using a bucket filled with water in a trial comparing exercise to a wait-and-see approach. The exercise protocol used concentric and eccentric wrist extension with progressive load, starting with 2kg for males and 1kg for females. Patients performed three sets of 15 repetitions daily and increased the load by 0.1kg each week for three months. Patients in the exercise group had significantly better outcomes than wait-and-see at three-month follow-up. The same authors then performed a second study of 120 patients splitting the protocol into eccentric exercise only versus concentric exercise only.[107] The eccentric exercise group achieved a faster and greater improvement in pain.

Selvanetti et al[108] used a home exercise combination of contract/relax stretching and eccentric loading of the wrist extensors in a trial against a control intervention of ultrasound and advice. Only the abstract is available in English, but at minimum six-month follow-up a large treatment effect was seen in the exercise group, significantly greater than controls.

Barratt et al[109] conducted a large service improvement project involving 182 patients. Firstly, usual care was assessed before a shift of focus was made towards strengthening exercises and finally a specific progressive loading protocol implemented. The protocol began with moderate to high load isometric exercises progressing to concentric and eccentric exercises with increasing load. Although the study was limited by its non-randomised design and loss to follow-up there was evidence that the specific progressive loading protocol was more effective than other care with the difference attributed to the higher load progressions of the specific protocol. Indeed, a systematic review of tendon adaption to loading concluded that it was the progression to high load exercise that is the key factor in stimulating a tissue response rather than the type of muscle contraction used during exercise, though this review only included studies of lower limb tendinopathy.[110]

Exercise dosing

Raman et al[85] conducted a review of the literature in 2012 regarding the choice of exercise and dosing used to treat LET. The findings demonstrated great heterogeneity in numbers of repetitions, sets of exercises, frequency of exercise and duration of the exercise course with no clear conclusion on the optimum level. In a more recent 2020 review focussed upon eccentric exercise only, Chen et al[101] found that exercises were typically performed in three sets of 10 to 15 repetitions separated by 30 seconds to a minute’s rest between each set. Exercise frequency ranged from three days per week to daily and the duration of treatment from three weeks to 12 weeks. Based upon theoretical healing times for tendon pathology and assessment of treatment effect size of high dose versus low

dose trials the authors' recommendation was to perform eccentric exercises at least once per day, in three sets of 10-15 repetitions, for a minimum period of six weeks.

Painful versus pain-free exercise

A systematic review of pain-free exercises versus exercises that allowed some level of pain, published in 2017, found a short-term benefit in favour of painful exercises up to three months.[111] The review does not contain any trials related to LET but six of the nine included trials related to tendinopathy so the findings may be transferrable. Pain-related fear can lead to central sensitisation of the nervous system resulting in higher perceived pain levels, so an exercise approach that focusses on avoiding pain may exacerbate this response.[112] Central sensitisation is a common feature in patients with LET, as identified by 10 studies included in a recent systematic review so needs to be considered in any intervention design.[113] Methods of addressing central sensitisation and pain-related fear have been proposed for clinical practice and can be applied to exercise interventions for LET.[112,114] These include education of the patient, addressing anxiety related to activity or exercise to reduce the threat response and graded exposure to painful activities. The Stasinopoulos protocol permits mild pain during exercise below 4/10 on a numerical rating scale (NRS) and includes graded exposure to a painful stimulus (loading of wrist extension using a weight) with gradual progression of increasing load. It was consistently effective in treating LET in seven trials, so might be a basis of this theory if applied to practice with additional patient education.[26-28,88-91]

Exercise Summary

Eccentric loading is the most frequently studied form of exercise for LET and appears effective, with some certainty in the short-term based upon trials of moderate quality. There is additional evidence for the supplementation of eccentric loading with isometric and concentric exercises to amplify the effect. Based upon modern understanding of pain science and previous trials involving pain-provoking exercise there is justification to encourage exercise into low levels of pain if supported by appropriate patient education.

Corticosteroid injections

The use of corticosteroid injection (CSI) to treat patients with LET is controversial with calls to stop made as long ago as 2010.[115] Despite this, a survey conducted in 2011 still showed that 48% of UK specialist clinicians used CSI as a primary treatment.[116] Whilst this number had declined in a similar UK survey conducted in 2017, 36% of respondents still used CSI as a first or second-line treatment.[38] The controversy stems from the conclusions of several large randomised controlled trials that showed worse outcomes at one year follow-up compared to patients treated without CSI.[2,4,23] Numerous studies consistently showed a significant reduction in pain up to six weeks following CSI with a large effect size.[117] This significant short-term effect may be attractive to patients as it can provide fast alleviation of symptoms and allow early return to work but the longer-term implications need to be considered. Mardaini-Kivi et al[118] found that the symptoms of 34.7% of patients had already returned 12 weeks after CSI. Bisset et al[2] compared CSI to multimodal physiotherapy or a wait-and-see approach that included general advice. At six weeks, CSI produced the greatest improvement but by 12 months had the worst outcome, even when compared to wait-and-see. The CSI group had a 72% recurrence rate at 12 months compared to just 8% with physiotherapy and 9% with wait-and-see. Coombes et al[23] compared CSI with a saline placebo injection and multimodal physiotherapy versus no physiotherapy in a 2 x 2 factorial design study. The two CSI groups showed the greatest improvements at four weeks but the worst outcomes at 12 months, even when

compared to the placebo injection and no physiotherapy. The recurrence rate at 12 months was 54% across the CSI groups. A subsequent economic evaluation from the same study concluded that CSI was not a cost-effective treatment for LET.[98] Smidt et al[4] compared CSI to multimodal physiotherapy or a wait-and-see approach. Again, CSI produced the greatest improvement at four weeks but by 12 weeks was no better than wait-and-see. At six months and one year the outcomes for those patients receiving CSI were worse than wait-and-see. Of the large randomised controlled studies of CSI for LET, it is only Hay et al[119] and Olaussen et al[6] that did not show a detrimental effect at one year follow-up. Hay et al[119] compared CSI to naproxen tablets or placebo vitamin C tablets. Olaussen et al[6] compared CSI plus multimodal physiotherapy with a placebo injection plus multimodal physiotherapy and a third wait-and-see group. By 12 months all groups had achieved a similar outcome but after an initially favourable response the CSI plus physiotherapy group had worse outcomes between 12 to 26 weeks compared to the other groups. Overall, the evidence would suggest therefore that CSI should be used with caution as despite strong evidence of short-term beneficial effect, the medium-term and long-term effect may be negative.

Multimodal physiotherapy

Many studies use a combination of treatments as part of a multimodal package of physiotherapy treatment. In particular, there are five large randomised trials totalling 845 patients, four of which had wait-and-see control groups, that have investigated a multimodal approach with a long-term follow-up of one year.[2,4,6,23,97] Three of these trials used the same multimodal approach proposed by Vicenzino in 2003.[2,23,96,97] Patients were educated regarding avoiding painful activities involving repetitive activity or gripping with the forearm pronated and elbow extended. A trial of MWM and taping was performed to establish if there is an immediate reduction in pain on gripping and patients were taught an exercise routine of posture correction, progressive forearm strengthening and general upper limb strengthening. Patients were then seen eight times over six to eight weeks in two trials[2,23] and four times over four weeks in one trial.[97] At these visits MWM and taping was repeated if found to be beneficial and the exercises were repeated under supervision and progressed as able. Exercises were continued at home. All three trials found significant short-term improvement with multimodal physiotherapy between four to six-week follow-up compared in two trials to a control of wait-and-see, and in one trial to prolotherapy. Additionally, Coombes et al[23] found multimodal physiotherapy superior to wait-and-see at 26 weeks and Yelland et al[97] superior to prolotherapy at 12 weeks. All three studies found that by 12 months the difference between control or prolotherapy was no-longer significant due to the fact that LET symptoms tend to improve in the majority of patients over time. Bisset et al[2], though, performed an area under the curve analysis to evaluate that, compared to CSI or a control of wait-and-see, multimodal physiotherapy was superior. It was also associated with the lowest symptom recurrence rate and lowest analgesic use.

Olaussen et al[6] compared multimodal physiotherapy with CSI or placebo injection against a control group of wait-and-see. The multimodal physiotherapy consisted of six sessions over six weeks of Cyriax manual therapy, passive stretches of the forearm extensor muscles and a home exercise programme of forearm extensor muscle stretching and eccentric strengthening. The wait-and-see group were given education regarding activity modification and were prescribed NSAIDs. At six-week follow-up multimodal physiotherapy was superior to wait-and-see but at subsequent assessments at 12, 26 and 52 weeks there was no difference between groups.

Smidt et al[4] compared multimodal physiotherapy against CSI and a control group of wait-and-see. The multimodal approach consisted of ultrasound, deep transverse friction massage and the

Pienimaki exercise programme of stretching and progressive strengthening for six weeks.[86] The highest probability of recovery at six-month follow-up was found in the multimodal physiotherapy group. At 12-months the success rate of the CSI group was 69% compared with 91% and 88% respectively in the multimodal physiotherapy and wait-and-see groups.

Overall, the evidence would suggest a positive short and mid-term effect of multimodal physiotherapy compared with control or comparator treatments but the key components of an optimum multimodal physiotherapy treatment package have not been established.

Summary

A wide range of treatment techniques have been investigated for LET. There is low or very low-quality evidence to suggest that manual therapy, laser, acupuncture, diamond taping and orthotics may give a short-term beneficial effect but the practicalities of using such interventions in a publicly-funded health service are questionable. There are uncertainties regarding the value of treatments that require numerous patient attendances, such as manual therapy, laser, taping or acupuncture. Exercise is supported by a greater evidence base but questions remain as to the optimum exercise choice and exercise dose. Stretching and eccentric exercise show beneficial effects but with the potential for further improvement with the addition of isometric and concentric exercises. Modern understanding of pain theory would suggest that exercise into mild pain might also improve outcomes. Trials have shown that many patients improve with simple advice and time, but there is potential to improve this self-management support further with the addition of psychological and behavioural interventions to improve patient self-efficacy.

Given the current lack of a consistent treatment approach provided in the UK and lack of certainty from the evidence base to guide clinicians, it is necessary to ascertain from clinical, managerial and patient stakeholders which are the most practical treatments for use in UK NHS practice as part of an optimised physiotherapy treatment package.

References:

1. National Institute for Health and Care Excellence. Clinical Knowledge Summaries: Tennis Elbow. https://cks.nice.org.uk/topics/tennis-elbow/management/management/. Accessed 16th September 2020.
2. Bisset L, Beller E, Jull G, Brooks P, Darnell R, Vicenzino B. Mobilisation with movement and exercise, corticosteroid injection, or wait and see for tennis elbow: randomised trial. *BMJ.* 2006;333(7575):939.
3. Peterson M, Butler S, Eriksson M, Svardsudd K. A randomized controlled trial of exercise versus wait-list in chronic tennis elbow (lateral epicondylosis). *Ups J Med Sci.* 2011;116(4):269-279.
4. Smidt N, van der Windt DAWM, Assendelft WJJ, Devillé WLJM, Korthals-de Bos IBC, Bouter LM. Corticosteroid injections, physiotherapy, or a wait-and-see policy for lateral epicondylitis: a randomised controlled trial. *The Lancet.* 2002;359(9307):657-662.
5. Vuvan V, Vicenzino B, Mellor R, Heales LJ, Coombes BK. Unsupervised Isometric Exercise versus Wait-and-See for Lateral Elbow Tendinopathy. *Med Sci Sports Exerc.* 2020;52(2):287-295.

6. Olaussen M, Holmedal O, Mdala I, Brage S, Lindbaek M. Corticosteroid or placebo injection combined with deep transverse friction massage, Mills manipulation, stretching and eccentric exercise for acute lateral epicondylitis: a randomised, controlled trial. *BMC Musculoskelet Disord.* 2015;16(1):122.
7. Shiri R, Viikari-Juntura E, Varonen H, Heliovaara M. Prevalence and determinants of lateral and medial epicondylitis: a population study. *Am J Epidemiol.* 2006;164(11):1065-1074.
8. Titchener AG, Fakis A, Tambe AA, Smith C, Hubbard RB, Clark DI. Risk factors in lateral epicondylitis (tennis elbow): a case-control study. *Journal of Hand Surgery (European Volume).* 2012;38(2):159-164.
9. Naylor C, Imison C, Addicott R, et al. *Transforming our healthcare system - Ten priorities for commissioners.* London: The Kings Fund; 2015.
10. Johnston V, Jull G, Sheppard DM, Ellis N. Applying principles of self-management to facilitate workers to return to or remain at work with a chronic musculoskeletal condition. *Man Ther.* 2013;18(4):274-280.
11. Babatunde OO, Jordan JL, Van Der Windt DA, Hill JC, Foster NE, Protheroe J. Effective treatment options for musculoskeletal pain in primary care: A systematic overview of current evidence. *PLoS One.* 2017;12(6):e0178621.
12. Carnes D, Homer KE, Miles CL, et al. Effective Delivery Styles and Content for Self-management Interventions for Chronic Musculoskeletal Pain: A Systematic Literature Review. *The Clinical Journal of Pain.* 2012;28(4).
13. Miles CL, Pincus T, Carnes D, et al. Can we identify how programmes aimed at promoting self-management in musculoskeletal pain work and who benefits? A systematic review of sub-group analysis within RCTs. *Eur J Pain.* 2011;15(8):775 e771-711.
14. Dineen-Griffin S, Garcia-Cardenas V, Williams K, Benrimoj SI. Helping patients help themselves: A systematic review of self-management support strategies in primary health care practice. *PLoS One.* 2019;14(8):e0220116.
15. Hutting N, Johnston V, Staal JB, Heerkens YF. Promoting the Use of Self-management Strategies for People With Persistent Musculoskeletal Disorders: The Role of Physical Therapists. *J Orthop Sports Phys Ther.* 2019;49(4):212-215.
16. Hatch GF, 3rd, Pink MM, Mohr KJ, Sethi PM, Jobe FW. The effect of tennis racket grip size on forearm muscle firing patterns. *Am J Sports Med.* 2006;34(12):1977-1983.
17. Mohandhas BR, Makaram N, Drew TS, Wang W, Arnold GP, Abboud RJ. Racquet string tension directly affects force experienced at the elbow: implications for the development of lateral epicondylitis in tennis players. *Shoulder & Elbow.* 2016;8(3):184-191.
18. King MA, Kentel BB, Mitchell SR. The effects of ball impact location and grip tightness on the arm, racquet and ball for one-handed tennis backhand groundstrokes. *J Biomech.* 2012;45(6):1048-1052.
19. Pattanittum P, Turner T, Green S, Buchbinder R. Non-steroidal anti-inflammatory drugs (NSAIDs) for treating lateral elbow pain in adults. *The Cochrane database of systematic reviews.* 2013(5):CD003686.
20. Lucado AM, Dale RB, Vincent J, Day JM. Do joint mobilizations assist in the recovery of lateral elbow tendinopathy? A systematic review and meta-analysis. *J Hand Ther.* 2019;32(2):262-276 e261.
21. Nagrale AV, Herd CR, Ganvir S, Ramteke G. Cyriax physiotherapy versus phonophoresis with supervised exercise in subjects with lateral epicondylalgia: a randomized clinical trial. *J Man Manip Ther.* 2009;17(3):171-178.
22. Vicenzino B, Paungmali A, Buratowski S, Wright A. Specific manipulative therapy treatment for chronic lateral epicondylalgia produces uniquely characteristic hypoalgesia. *Man Ther.* 2001;6(4):205-212.

23. Coombes BK, Bisset L, Brooks P, Khan A, Vicenzino B. Effect of corticosteroid injection, physiotherapy, or both on clinical outcomes in patients with unilateral lateral epicondylalgia: A randomized controlled trial. *JAMA.* 2013;309(5):461-469.
24. Amro A, Diener I, Bdair WO, Hameda IM, Shalabi AI, Ilyyan DI. The effects of Mulligan mobilisation with movement and taping techniques on pain, grip strength, and function in patients with lateral epicondylitis. *Hong Kong Physiotherapy Journal.* 2010;28(1):19-23.
25. Kim LJ, Choi H, Moon D. Improvement of Pain and Functional Activities in Patients with Lateral Epicondylitis of the Elbow by Mobilization with Movement: a Randomized, Placebo-Controlled Pilot Study. *Journal of Physical Therapy Science.* 2012;24(9):787-790.
26. Stasinopoulos D, Stasinopoulos I. Comparison of effects of Cyriax physiotherapy, a supervised exercise programme and polarized polychromatic non-coherent light (Bioptron light) for the treatment of lateral epicondylitis. *Clin Rehabil.* 2006;20(1):12-23.
27. Viswas R, Ramachandran R, Korde Anantkumar P. Comparison of effectiveness of supervised exercise program and Cyriax physiotherapy in patients with tennis elbow (lateral epicondylitis): a randomized clinical trial. *ScientificWorldJournal.* 2012;2012:939645.
28. Stasinopoulos D, Stasinopoulou K, Johnson MI. An exercise programme for the management of lateral elbow tendinopathy. *Br J Sports Med.* 2005;39(12):944-947.
29. Paungmali A, O'Leary S, Souvlis T, Vicenzino B. Hypoalgesic and Sympathoexcitatory Effects of Mobilization With Movement for Lateral Epicondylalgia. *Phys Ther.* 2003;83(4):374-383.
30. Afzal MW, Ahmad A, Waqas MS, Ahmad U. Effectiveness of Therapeutic Ultrasound with and without Mulligan Mobilzation in Lateral Epicondylitis. *Annals of King Edward Medical University.* 2016;22(1):47.
31. Martinez-Cervera FV, Olteanu TE, Gil-Martinez A, Diaz-Pulido B, Ferrer-Pena R. Influence of expectations plus mobilization with movement in patient with lateral epicondylalgia: a pilot randomized controlled trial. *J Exerc Rehabil.* 2017;13(1):101-109.
32. Joshi S, Metgud S, Ebnezer C. Comparing the effects of manipulation of wrist and ultrasound, friction massage and exercises on lateral epicondylitis: a randomized clinical study. *Indian Journal of Physiotherapy and Occupational Therapy.* 2013;7(3):205.
33. Struijs PAA, Damen P-J, Bakker EWP, Blankevoort L, Assendelft WJJ, van Dijk CN. Manipulation of the Wrist for Management of Lateral Epicondylitis: A Randomized Pilot Study. *Phys Ther.* 2003;83(7):608-616.
34. Cleland JA, Whitman JM, Fritz JM. Effectiveness of manual physical therapy to the cervical spine in the management of lateral epicondylalgia: a retrospective analysis. *J Orthop Sports Phys Ther.* 2004;34(11):713-722; discussion 722-714.
35. Fernández-Carnero J, Cleland JA, Arbizu RL. Examination of motor and hypoalgesic effects of cervical vs thoracic spine manipulation in patients with lateral epicondylalgia: a clinical trial. *J Manipulative Physiol Ther.* 2011;34(7):432-440.
36. Fernandez-Carnero J, Fernandez-de-las-Penas C, Cleland JA. Immediate hypoalgesic and motor effects after a single cervical spine manipulation in subjects with lateral epicondylalgia. *J Manipulative Physiol Ther.* 2008;31(9):675-681.
37. Vicenzino B, Collins D, Wright A. The initial effects of a cervical spine manipulative physiotherapy treatment on the pain and dysfunction of lateral epicondylalgia. *Pain.* 1996;68(1):69-74.
38. Bateman M, Titchener AG, Clark DI, Tambe AA. Management of tennis elbow: a survey of UK clinical practice. *Shoulder Elbow.* 2019;11(3):233-238.
39. Meyer NJ, Pennington W, Haines B, Daley R. The effect of the forearm support band on forces at the origin of the extensor carpi radialis brevis: a cadaveric study and review of literature. *J Hand Ther.* 2002;15(2):179-184.
40. Kroslak M, Pirapakaran K, Murrell GAC. Counterforce bracing of lateral epicondylitis: a prospective, randomized, double-blinded, placebo-controlled clinical trial. *J Shoulder Elbow Surg.* 2019;28(2):288-295.

41. Barati H, Zarezadeh A, MacDermid JC, Sadeghi-Demneh E. The immediate sensorimotor effects of elbow orthoses in patients with lateral elbow tendinopathy: a prospective crossover study. *J Shoulder Elbow Surg.* 2019;28(1):e10-e17.
42. Kachanathu SJ, Alenazi AM, Hafez AR, Algarni AD, Alsubiheen AM. Comparison of the effects of short-duration wrist joint splinting combined with physical therapy and physical therapy alone on the management of patients with lateral epicondylitis. *Eur J Phys Rehabil Med.* 2019;55(4):488-493.
43. Akkurt HE, Kocabas H, Yilmaz H, et al. Comparison of an epicondylitis bandage with a wrist orthosis in patients with lateral epicondylitis. *Prosthet Orthot Int.* 2018;42(6):599-605.
44. Garg R, Adamson GJ, Dawson PA, Shankwiler JA, Pink MM. A prospective randomized study comparing a forearm strap brace versus a wrist splint for the treatment of lateral epicondylitis. *J Shoulder Elbow Surg.* 2010;19(4):508-512.
45. Van De Streek MD, Van Der Schans CP, De Greef MH, Postema K. The effect of a forearm/hand splint compared with an elbow band as a treatment for lateral epicondylitis. *Prosthet Orthot Int.* 2004;28(2):183-189.
46. Nishizuka T, Iwatsuki K, Kurimoto S, Yamamoto M, Hirata H. Efficacy of a forearm band in addition to exercises compared with exercises alone for lateral epicondylitis: A multicenter, randomized, controlled trial. *J Orthop Sci.* 2017;22(2):289-294.
47. Solveborn SA. Radial epicondylalgia ('tennis elbow'): treatment with stretching or forearm band. A prospective study with long-term follow-up including range-of-motion measurements. *Scand J Med Sci Sports.* 1997;7(4):229-237.
48. Coombes BK, Bisset L, Vicenzino B. Management of Lateral Elbow Tendinopathy: One Size Does Not Fit All. *J Orthop Sports Phys Ther.* 2015;45(11):938-949.
49. Wegener RL, Brown T, O'Brien L. A randomized controlled trial of comparative effectiveness of elastic therapeutic tape, sham tape or eccentric exercises alone for lateral elbow tendinosis. *Hand Therapy.* 2016;21(4):131-139.
50. Cho YT, Hsu WY, Lin LF, Lin YN. Kinesio taping reduces elbow pain during resisted wrist extension in patients with chronic lateral epicondylitis: a randomized, double-blinded, cross-over study. *BMC Musculoskelet Disord.* 2018;19(1):193.
51. Au IPH, Fan PCP, Lee WY, et al. Effects of Kinesio tape in individuals with lateral epicondylitis: A deceptive crossover trial. *Physiother Theory Pract.* 2017;33(12):914-919.
52. Dilek B, Batmaz I, Sariyildiz MA, et al. Kinesio taping in patients with lateral epicondylitis. *J Back Musculoskelet Rehabil.* 2016;29(4):853-858.
53. Dones VC, 3rd, Serra MAB, Kamus GOT, 3rd, et al. The effectiveness of Biomechanical Taping Technique on visual analogue scale, static maximum handgrip strength, and Patient Rated Tennis Elbow Evaluation of patients with lateral epicondylalgia: A cross-over study. *J Bodyw Mov Ther.* 2019;23(2):405-416.
54. Eraslan L, Yuce D, Erbilici A, Baltaci G. Does Kinesiotaping improve pain and functionality in patients with newly diagnosed lateral epicondylitis? *Knee Surg Sports Traumatol Arthrosc.* 2018;26(3):938-945.
55. Giray E, Karali-Bingul D, Akyuz G. The Effectiveness of Kinesiotaping, Sham Taping or Exercises Only in Lateral Epicondylitis Treatment: A Randomized Controlled Study. *PM R.* 2019;11(7):681-693.
56. Vicenzino B, Brooksbank J, Minto J, Offord S, Paungmali A. Initial effects of elbow taping on pain-free grip strength and pressure pain threshold. *J Orthop Sports Phys Ther.* 2003;33(7):400-407.
57. George CE, Heales LJ, Stanton R, Wintour SA, Kean CO. Sticking to the facts: A systematic review of the effects of therapeutic tape in lateral epicondylalgia. *Phys Ther Sport.* 2019;40:117-127.

58. Gadau M, Yeung WF, Liu H, et al. Acupuncture and moxibustion for lateral elbow pain: a systematic review of randomized controlled trials. *BMC Complement Altern Med.* 2014;14(1):136.
59. Jeon J, Bussin E, Scott A. Temporal divergence of changes in pain and pain-free grip strength after manual acupuncture or electroacupuncture: an experimental study in people with lateral epicondylalgia. *Chin Med.* 2017;12:22.
60. Bisset L, Coombes B, Vicenzino B. Tennis elbow. *BMJ Clin Evid.* 2011;2011.
61. Long L, Briscoe S, Cooper C, Hyde C, Crathorne L. What is the clinical effectiveness and cost-effectiveness of conservative interventions for tendinopathy? An overview of systematic reviews of clinical effectiveness and systematic review of economic evaluations. *Health Technol Assess.* 2015;19(8):1-134.
62. Tang H, Fan H, Chen J, et al. Acupuncture for Lateral Epicondylitis: A Systematic Review. *Evid Based Complement Alternat Med.* 2015;2015:861849.
63. Fink M, Wolkenstein E, Luennemann M, Gutenbrunner C, Gehrke A, Karst M. Chronic epicondylitis: effects of real and sham acupuncture treatment: a randomised controlled patient- and examiner-blinded long-term trial. *Forsch Komplementarmed Klass Naturheilkd.* 2002;9(4):210-215.
64. Irnich D, Karg H, Behrens N, et al. Controlled Trial on Point Specificity of Acupuncture in the Treatment of Lateral Epicondylitis (Tennis Elbow). *Physikalische Medizin, Rehabilitationsmedizin, Kurortmedizin.* 2003;13(04):215-219.
65. Haker E, Lundeberg T. Acupuncture treatment in epicondylalgia: a comparative study of two acupuncture techniques. *Clin J Pain.* 1990;6(3):221-226.
66. Molsberger A, Hille E. The analgesic effect of acupuncture in chronic tennis elbow pain. *Br J Rheumatol.* 1994;33(12):1162-1165.
67. Basford JR, Sheffield CG, Cieslak KR. Laser therapy: A randomized, controlled trial of the effects of low intensity Nd:YAG laser irradiation on lateral epicondylitis. *Arch Phys Med Rehabil.* 2000;81(11):1504-1510.
68. Carulli C, Tonelli F, Innocenti M, Gambardella B, Muncibi F, Innocenti M. Effectiveness of extracorporeal shockwave therapy in three major tendon diseases. *J Orthop Traumatol.* 2016;17(1):15-20.
69. D'Vaz AP, Ostor AJ, Speed CA, et al. Pulsed low-intensity ultrasound therapy for chronic lateral epicondylitis: a randomized controlled trial. *Rheumatology (Oxford).* 2006;45(5):566-570.
70. Buchbinder R, Green SE, Youd JM, Assendelft WJJ, Barnsley L, Smidt N. Systematic review of the efficacy and safety of shock wave therapy for lateral elbow pain. *The Journal of Rheumatology.* 2006;33(7):1351.
71. Dingemanse R, Randsdorp M, Koes BW, Huisstede BM. Evidence for the effectiveness of electrophysical modalities for treatment of medial and lateral epicondylitis: a systematic review. *Br J Sports Med.* 2014;48(12):957-965.
72. Bateman M, Whitby E, Kacha S, Salt E. Current physiotherapy practice in the management of tennis elbow: A service evaluation. *Musculoskeletal Care.* 2018;16(2):322-326.
73. Bjordal JM, Lopes-Martins RA, Joensen J, et al. A systematic review with procedural assessments and meta-analysis of low level laser therapy in lateral elbow tendinopathy (tennis elbow). *BMC Musculoskelet Disord.* 2008;9(1):75.
74. Kraushaar BS, Nirschl RP. Current Concepts Review - Tendinosis of the Elbow (Tennis Elbow). Clinical Features and Findings of Histological, Immunohistochemical, and Electron Microscopy Studies*. *JBJS.* 1999;81(2).
75. Haker EH, Lundeberg TC. Lateral epicondylalgia: report of noneffective midlaser treatment. *Arch Phys Med Rehabil.* 1991;72(12):984-988.
76. Papadopoulos ES, Smith RW, Cawley M, Mani R. Low-level laser therapy does not aid the management of tennis elbow. *Clin Rehabil.* 1996;10(1):9-11.

77. Lögdberg-Andersson M, Mützell S, Hazel Å. Low Level Laser Therapy (Lllt) of Tendinitis and Myofascial Pains a Randomized, Double-Blind, Controlled Study. *Laser Therapy.* 2004;14(0_Pilot_Issue_2):0_79-70_84.
78. Shah SGS, Farrow A. Trends in the availability and usage of electrophysical agents in physiotherapy practices from 1990 to 2010: a review. *Phys Ther Rev.* 2013;17(4):207-226.
79. Bisset L, Paungmali A, Vicenzino B, Beller E. A systematic review and meta-analysis of clinical trials on physical interventions for lateral epicondylalgia. *Br J Sports Med.* 2005;39(7):411-422; discussion 411-422.
80. Oken O, Kahraman Y, Ayhan F, Canpolat S, Yorgancioglu ZR, Oken OF. The short-term efficacy of laser, brace, and ultrasound treatment in lateral epicondylitis: a prospective, randomized, controlled trial. *J Hand Ther.* 2008;21(1):63-67; quiz 68.
81. Smidt N, Assendelft WJ, Arola H, et al. Effectiveness of physiotherapy for lateral epicondylitis: a systematic review. *Ann Med.* 2003;35(1):51-62.
82. Binder A, Hodge G, Greenwood AM, Hazleman BL, Page Thomas DP. Is therapeutic ultrasound effective in treating soft tissue lesions? *Br Med J (Clin Res Ed).* 1985;290(6467):512-514.
83. Lundeberg T, Abrahamsson P, Haker E. A comparative study of continuous ultrasound, placebo ultrasound and rest in epicondylalgia. *Scand J Rehabil Med.* 1988;20(3):99-101.
84. Bisset LM, Vicenzino B. Physiotherapy management of lateral epicondylalgia. *J Physiother.* 2015;61(4):174-181.
85. Raman J, MacDermid JC, Grewal R. Effectiveness of different methods of resistance exercises in lateral epicondylosis--a systematic review. *J Hand Ther.* 2012;25(1):5-25.
86. Pienimäki TT, Tarvainen TK, Siira PT, Vanharanta H. Progressive Strengthening and Stretching Exercises and Ultrasound for Chronic Lateral Epicondylitis. *Physiotherapy.* 1996;82(9):522-530.
87. Tonks JH, Pai SK, Murali SR. Steroid injection therapy is the best conservative treatment for lateral epicondylitis: a prospective randomised controlled trial. *Int J Clin Pract.* 2007;61(2):240-246.
88. Manias P, Stasinopoulos D. A controlled clinical pilot trial to study the effectiveness of ice as a supplement to the exercise programme for the management of lateral elbow tendinopathy. *Br J Sports Med.* 2006;40(1):81-85.
89. Sethi K, Noohu MM. Scapular muscles strengthening on pain, functional outcome and muscle activity in chronic lateral epicondylalgia. *J Orthop Sci.* 2018;23(5):777-782.
90. Stasinopoulos D, Manias P. Comparing Two Exercise Programmes for the Management of Lateral Elbow Tendinopathy (Tennis Elbow/Lateral Epicondylitis)-A Controlled Clinical Trial. *The Open Access Journal of Science and Technology.* 2013;1.
91. Stasinopoulos D, Stasinopoulos I. Comparison of effects of eccentric training, eccentric-concentric training, and eccentric-concentric training combined with isometric contraction in the treatment of lateral elbow tendinopathy. *J Hand Ther.* 2017;30(1):13-19.
92. Mostafaee N, Divandari A, Negahban H, et al. Shoulder and scapula muscle training plus conventional physiotherapy versus conventional physiotherapy only: a randomized controlled trial of patients with lateral elbow tendinopathy. *Physiother Theory Pract.* 2020:1-12.
93. Haahr JP, Andersen JH. Prognostic factors in lateral epicondylitis: a randomized trial with one-year follow-up in 266 new cases treated with minimal occupational intervention or the usual approach in general practice. *Rheumatology (Oxford).* 2003;42(10):1216-1225.
94. Nilsson P, Thom E, Baigi A, Marklund B, Månsson J. A prospective pilot study of a multidisciplinary home training programme for lateral epicondylitis. 2007;5:36-50.
95. Svernlöv B, Adolfsson L. Non-operative treatment regime including eccentric training for lateral humeral epicondylalgia. *Scand J Med Sci Sports.* 2001;11(6):328-334.

96. Vicenzino B. Lateral epicondylalgia: a musculoskeletal physiotherapy perspective. *Man Ther.* 2003;8(2):66-79.
97. Yelland M, Rabago D, Ryan M, et al. Prolotherapy injections and physiotherapy used singly and in combination for lateral epicondylalgia: a single-blinded randomised clinical trial. *BMC Musculoskelet Disord.* 2019;20(1):509.
98. Coombes BK, Connelly L, Bisset L, Vicenzino B. Economic evaluation favours physiotherapy but not corticosteroid injection as a first-line intervention for chronic lateral epicondylalgia: evidence from a randomised clinical trial. *Br J Sports Med.* 2016;50(22):1400-1405.
99. Silbernagel KG, Vicenzino BT, Rathleff MS, Thorborg K. Isometric exercise for acute pain relief: is it relevant in tendinopathy management? *Br J Sports Med.* 2019;53(21):1330-1331.
100. Park JY, Park HK, Choi JH, et al. Prospective evaluation of the effectiveness of a home-based program of isometric strengthening exercises: 12-month follow-up. *Clin Orthop Surg.* 2010;2(3):173-178.
101. Chen Z, Baker NA. Effectiveness of eccentric strengthening in the treatment of lateral elbow tendinopathy: A systematic review with meta-analysis. *J Hand Ther.* 2020.
102. Tyler TF, Thomas GC, Nicholas SJ, McHugh MP. Addition of isolated wrist extensor eccentric exercise to standard treatment for chronic lateral epicondylosis: a prospective randomized trial. *J Shoulder Elbow Surg.* 2010;19(6):917-922.
103. Tiwari M. Effectiveness of Flex Bar Eccentric Exercises Versus Progressive Resistance Exercises of Wrist on Pain, Pain Free Grip Strength and Functional Activities in Sub Acute Tennis Elbow. *Indian Journal of Physiotherapy & Occupational Therapy.* 2018;12(3).
104. Martinez-Silvestrini JA, Newcomer KL, Gay RE, Schaefer MP, Kortebein P, Arendt KW. Chronic lateral epicondylitis: comparative effectiveness of a home exercise program including stretching alone versus stretching supplemented with eccentric or concentric strengthening. *J Hand Ther.* 2005;18(4):411-419, quiz 420.
105. Soderberg J, Grooten WJ, Ang BO. Effects of eccentric training on hand strength in subjects with lateral epicondylalgia: a randomized-controlled trial. *Scand J Med Sci Sports.* 2012;22(6):797-803.
106. Croisier JL, Foidart-Dessalle M, Tinant F, Crielaard JM, Forthomme B. An isokinetic eccentric programme for the management of chronic lateral epicondylar tendinopathy. *Br J Sports Med.* 2007;41(4):269-275.
107. Peterson M, Butler S, Eriksson M, Svardsudd K. A randomized controlled trial of eccentric vs. concentric graded exercise in chronic tennis elbow (lateral elbow tendinopathy). *Clin Rehabil.* 2014;28(9):862-872.
108. Selvanetti A, Barrucci A, Antonaci A, MARTINEZI P, Marra S, Necozione S. Role of the eccentric exercise in the functional reeducation of lateral epicondylitis: a randomised controlled clinical trial. *Med Sport.* 2003;56:103-113.
109. Barratt PA, Selfe J. A Service Evaluation and Improvement Project: A Three Year Systematic Audit Cycle of the Physiotherapy Treatment for Lateral Epicondylalgia. *Physiotherapy.* 2017.
110. Bohm S, Mersmann F, Arampatzis A. Human tendon adaptation in response to mechanical loading: a systematic review and meta-analysis of exercise intervention studies on healthy adults. *Sports Medicine - Open.* 2015;1(1):7.
111. Smith BE, Hendrick P, Smith TO, et al. Should exercises be painful in the management of chronic musculoskeletal pain? A systematic review and meta-analysis. *Br J Sports Med.* 2017;51(23):1679-1687.
112. Smith BE, Hendrick P, Bateman M, et al. Musculoskeletal pain and exercise-challenging existing paradigms and introducing new. *Br J Sports Med.* 2019;53(14):907-912.
113. Plinsinga ML, Brink MS, Vicenzino B, van Wilgen CP. Evidence of Nervous System Sensitization in Commonly Presenting and Persistent Painful Tendinopathies: A Systematic Review. *J Orthop Sports Phys Ther.* 2015;45(11):864-875.

114. Nijs J, Lluch Girbés E, Lundberg M, Malfliet A, Sterling M. Exercise therapy for chronic musculoskeletal pain: Innovation by altering pain memories. *Man Ther.* 2015;20(1):216-220.
115. Osborne H. Stop injecting corticosteroid into patients with tennis elbow, they are much more likely to get better by themselves! *J Sci Med Sport.* 2010;13(4):380-381.
116. Titchener AG, Booker SJ, Bhamber NS, Tambe AA, Clark DI. Corticosteroid and platelet-rich plasma injection therapy in tennis elbow (lateral epicondylalgia): a survey of current U.K. specialist practice and a call for clinical guidelines. *Br J Sports Med.* 2015;49(21):1410-1413.
117. Olaussen M, Holmedal O, Lindbaek M, Brage S, Solvang H. Treating lateral epicondylitis with corticosteroid injections or non-electrotherapeutical physiotherapy: a systematic review. *BMJ Open.* 2013;3(10):e003564.
118. Mardani-Kivi M, Karimi-Mobarakeh M, Karimi A, et al. The effects of corticosteroid injection versus local anesthetic injection in the treatment of lateral epicondylitis: a randomized single-blinded clinical trial. *Arch Orthop Trauma Surg.* 2013;133(6):757-763.
119. Hay EM, Paterson SM, Lewis M, Hosie G, Croft P. Pragmatic randomised controlled trial of local corticosteroid injection and naproxen for treatment of lateral epicondylitis of elbow in primary care. *BMJ.* 1999;319(7215):964-968.
